# Dynamic Changes of IFN-γ-producing Cells, TGF-β and Their Preidctive Value in Early Outcomees of Renal Transplantation

**Published:** 2013-05-01

**Authors:** F. Mohammadi, M. H. Niknam, M. Nafar, B. Einollahi, B. Nazari, M. Lessanpezeshki, M. A. Amirzargar, G. Solgi, B. Nikbin, A. A. Amirzargar

**Affiliations:** 1 *Molecular Immunology Research Center and Department of Immunology, Faculty of Medicine, Tehran University of Medical Sciences, Tehran, Iran *; 2 *Department of Nephrology, Shahid Labbafinejad Hospital, Shahid Beheshti University of Medical Sciences, Tehran, Iran *; 3 *Nephrology Research Center, Faculty of Medicine, Baqiyatallah University of Medical Sciences, Tehran, Iran *; 4 *Nephrology Research Center, Faculty of Medicine, Tehran University of Medical Sciences, Tehran, Iran *; 5 *Department of Immunology, Faculty of Medicine, Hamadan University of Medical Sciences, Hamadan, Iran*

**Keywords:** Kidney, Allograft, TGF-β, IFN-γ

## Abstract

Background: A growing body of evidence demonstrated an immune etiology as well as nonimmune mechanisms for episodes of clinical acute rejection and long-term allograft dysfunction.

Objective: To investigate the correlation of IFN-γ-producing cells and TGF-β with incidence of clinical acute rejection in living-related and unrelated kidney allogarft recipients during the first post-transplant year.

Methods: This multi-center study was performed on 57 kidney allograft recipients from living-related (n=20) and unrelated (n=37) donors between April 2011 and September 2012 and who were followed prospectively for a mean period of one year. Peripheral blood samples were collected from all patients pre-transplantation and at days 14, 30 and 90 after transplantation; PBMCs were used as responding cells in enzyme-linked immunosorbent spot (ELISPOT) assay to measure the frequency of IFN-γ-producing cells after stimulation with donor lymphocytes. Additionally, TGF-β levels were measured in cell culture supernatants of ELISPOT assay.

Results: During the follow-up period, 45 (79%) patients were diagnosed with stable graft function (group A); 12 (21%) experienced clinical acute rejection episodes (group B). The frequency of IFN-γ-producing cells was significantly (p<0.001) higher in the rejection group in all three times after transplantation. Also, post-transplantation comparison for TGF-β showed a significantly (p<0.001) higher contents in group A vs. group B. Comparing the post-transplantation levels of TGF-β and mean numbers of IFN-γ- producing cells between groups A and B demonstrated a continuous increment in TGF-β and decreasing frequencies of IFN-γ-producing cells in group A vs. group B.

Conclusion: Serial post-transplantation monitoring of IFN-γ-producing donor reactive cells during the first months is a clinically feasible approach for identification of kidney allogarft recipients at risk for ongoing immune-mediated graft damage and later graft loss.

## INTRODUCTION

Agrowing body of evidence demonstrated an immune etiology as well as non-immune mechanisms for episodes of clinical acute rejection and long-term al lograft dysfunction [1, 2]. In spite of significant improvements in short-term and to a lesser extent, in long-term allograft survival, use of new immunosuppressive agents with their complications and adverse effects such as opportunistic infections, malignancies and cardiovascular diseases, still remains a life-threatening factor for allograft recipients [3, 4]. Therefore, a primary goal of transplant physicians is to minimize these complications using reliable markers to monitor the alloimmune responses that could provide a basis for individualized immunosuppressive treatment. Those immune markers allow to categorize transplant patients into high-risk and low-risk for immunological graft loss, which in turn allowing drug minimization in low-risk patients and early therapeutic interventions in high-risk patients [1, 3]. Because of a central role of T lymphocytes in alloreactive responses leading to both acute and chronic allograft rejection, measuring alloreactive T cell reactivity using a highly sensitive and reliable method, enzyme-linked immunosorbent spot (ELISPOT) assay, described for the first time by Heeger and colleagues [5], is of considerable interest to be defined as surrogate markers for long-term outcomes of allograft [1, 2]. Cytokine ELISPOT assay, particularly measuring the frequencies of IFN-γ-producing T cells before and after renal transplantation, as a predictor of post-transplantation outcomes, has been investigated in several previous studies [1, 2, 6-8]. 

Cytokines are potential immunomodulating molecules that play an important role in alloimmune responses against allografts and pathogens. The majority of cytokines are secreted by Th1, Th2, Th3, and Th17 cells which are effective in either allogarft acceptance or rejection [9-11]. 

It has been shown that IFN-γ secreted from NK cells, cytotoxic, and helper T cells is a potent stimulator of cell-mediated immune response leading to allograft rejection [12]. However, various studies concerning organ transplantation reflect distinct results. Paradoxically, in mouse model of kidney allogarft IFN-γ inhibited necrosis [13, 14], and IFN-γ or IFN-γ receptor deficiency enhances tissue necrosis. The mechanism is still unclear, nevertheless, it may remove the normal inhibitory effect of IFN-γ on CTLs and as a consequence, strengthen the Ab production [15, 16]. Other studies have shown that increased IFN-γ production elevates the risk of acute rejection in early post transplantation and within the first post-operative year [1, 17]. 

 Another cytokines which is mainly produced from Th2, Th3, and regulatory T cells inside the allogarft is TGF-β1, a pleiotropic cytokine that plays a role in biological processes such as angiogenesis and cell differentiation. TGF-β1 has been known as a tolerogenic cytokine which can convert CD4+ CD25+ T cells to the regulatory phenotypes [18]. Further more, TGF-β1 in kidney allograft recipients can act as profibrotic factor and consequently cause long-term allogarft dysfunction [19, 20]. On the other hand, significant reduction of TGF-β1 in renal transplant recipients is in favor of T cell activation and increasing risk of acute graft rejection [21]. 

In this prospective study, we used the ELISPOT method to measure the frequency of IFN-γ-prouducing cells among peripheral blood lymphocytes of kidney allograft recipients in response to stimulator cells from donors. Additionally, we measured the concentration of TGF-β1 in supernatants of ELISPOT cell cultures for evaluation of potential regulatory responses. In the next step, we investigated the correlation of these prognostic immune markers with the incidence of clinical acute rejection in living-related and unrelated kidney allogarft recipients during the first post-transplantation year. 

## PATIENTS AND METHODS

This prospective multicenter study was performed on 57 kidney allograft recipients with background diseases such as diabetes, hypertension, kidney stone and glomerulonephritis between April 2011 and September 2012 in three university hospitals (Imam Khomeini, Baghiatallah, and Labbafi Nedjad). The pa tients received either living-related (n=20) or unrelated (n=37) donor renal transplant and followed prospectively for a mean period of one year. Informed consent was obtained from the study subjects according to the protocols approved by the Tehran University of Medical Sciences Research Ethics Committee. Immunosuppressive regimen was instituted 24 hours before transplantation and the protocol consisted of adjusted dose of cyclosporine A, mycophenolate mofetil (MMF) and methylprednisolone. Pre-transplantation percentage of panel-reactive antibody (PRA) and WBC cross-match were performed by microlymphocytotoxicity test. HLA-DNA typing was done by standard PCR-SSP technique for all donors and recipients to determine the HLA-A, B and DRB1 alleles (HLA-A B DR Low Resolution kit, Biotest, Germany). 

Peripheral blood samples were collected from all patients pre-transplantation and on days 14, 30, and 90 post-transplantation to find the relationship between prognostic immunological markers (IFN-γ and TGF-β) and clinical acute rejection during the first year of the operation. Additionally, at the same time interval, 10 more renal transplantation patients who were diagnosed with biopsy-proven acute rejection (8 with acute cellular rejection Banff III; one with antibody-mediated rejection, Banff II; and one with acute cellular and humoral rejection, Banff IV) [22] within three months post-operatively were included as controls for this study. From the control group, blood samples was taken only after confirmation of acute rejection using biopsy protocol. 

Clinical follow-up

The primary outcome measures were the frequencies of acute rejection episodes, delayed graft function (DGF) and stable graft function. Clinical acute rejection was considered after an increase of creatinine by 0.3 mg/dL or greater from the baseline and confirmed by renal biopsy. Delayed graft function was defined as a requirement for dialysis within the first week post-transplantation because of rising serum creatinine after ruling out of other causes of graft dysfunction. Patients with no history of clinical and/or biopsy-proven rejection and with good functioning graft, as judged by serum creatinine level (<1.5 mg/ dL), were considered as stable graft function. Secondary end-points were incidence of CMV or other infections and need for hospitalization due to any reasons after discharge from hospital. 

Enzyme-linked immunosorbent spot (ELISPOT) assay 

Fresh peripheral blood mononuclear cells (PBMCs) were isolated from pre- and post-transplantation samples by standard Ficoll (Amersham, Germany) density gradient centrifugation and frozen until ELISPOT assay. The PBMCs were put in cryo tubes in the presence of DMSO and FBS (1:10 v/v) and kept at -20 °C for 2 hrs and at -70 °C for 24 hrs; the samples were then transferred to liquid nitrogen. At the time of making the assay, PBMCs were thawed in the presence of prewarmed RPMI-1640 medium and washed by incomplete RPMI-1640 (Gibco, Germany); thereafter, viable cells were enumerated by using trypan blue staining. 

In the next step, PBMCs were used as responding cells in ELISPOT assay to measure the frequency of IFN-γ-producing cells after stimulation with phytohemagglutinin (PHA) (GIBCO-mitogen) and donor lymphocytes. 

The ELISPOT method was used according to the manufacturer’s protocol (Human IFN-γ ELISPOT ready-set-go-eBioscience, Vienna, Austria). In brief, ELISPOT plates (MAIPS4510, Millipore) were coated with diluted capture antibodies against IFN-γ and incubated overnight at 4 °C. After washing, plates were blocked with RPMI 1640 containing L-glutamine plus penicillin/streptomycin for 1 h; then, additional washing was performed. Three wells in a duplicate were designated for each sample and 1×105 PBMCs in 100 μL of complete RPMI-1640 containing 10% human serum (Gibco) were added to each well. Thereafter, cells were tested against 4 μL PHA in two wells as positive controls, donor inactivated (by mitomycine, [Sigma-M0503]) lymphocytes (2×104 cells/well) in two wells as test, and finally negative control wells contain ing only recipient cells in RPMI-1640 without stimulation. After incubation at 37 °C and 5% CO2 for 48 hrs, the cells and medium were collected from the wells. The supernatant was used for measurement of TGF-β by ELISA method. Plates were washed with ELISPOT wash buffer; then the detection antibody was added. The plates were incubated at room temperature for 2 hrs and, after another washing step, horseradish peroxidase was added. Following incubation for 45 min at room temperature, 3-amino-9-ethylcarbazole (AEC) (Sigma-Aldrich) was applied to develop spots; the resulting spots were counted with the help of a dissecting microscope. Results were given as the frequencies of IFN-γ-producing cells. The mean number of IFN-γ spots per 1×105 PBMCs was calculated by subtracting the values measured in the test wells from that of the negative control well for each sample. 

TGF-β measurement by enzyme-linked immunosorbent assay (ELISA) 

This assay was carried out on the collected supernants from the ELISPOT plates (described above). The assay procedure was performed based on the manufacture’s instructions (Human/Mouse TGF-β ELISA ready-set-go-eBioscience, Vienna, Austria). Briefly, in the first step, diluted capture antibody in coating buffer was added to each well of a corning coaster NUNC Maxiorp 96-well plate. Then, the plate was covered and incubated overnight at 4 °C. Thereafter, aspiration of the antibody solution and washing was done and then 1X solution of assay diluent was added to each well and incubated at room temperature for 1 hr. Finally, by discarding the assay diluent and another washing step, the plate was used for TGF-β measurement. 

Sample preparation 

Before measuring TGF-β content in the supernatants, the latent form of this cytokine must be converted to immunoreactive form. For this purpose, 100 μL of the sample was mixed with 20 μL 1-N HCl and incubated at room temperature for 10 min and then neutralized by adding 20 μL of 1-N NaOH. For standard solutions, serial dilutions were prepared according to the kit’s protocol in order to draw standard curve for quantification of TGF-β levels. 

In the next step, 100 μL of each standard and prepared samples was added to the wells in duplicate; then, the plate was covered and incubated at room temperature for 2 hrs. Subsequently, detection antibody, AVi-HRP, substrate and stop solution were added to each well according to the kit’s instructions. Finally, the concentration of TGF-β in supernatants was determined using the provided standard curve from different standard solutions. 

Statistical analysis 

Data were presented as mean±SD or mean±SEM. Groups were compared using the χ2 or Fisher’s exact tests for categorical variables. The *Student’s t *test for normally distributed data and Mann-Whitney U test for nonparametric variables were used. Tests of within-subject effects, tests of between-subject effects, pairwise comparisons, ANOVA test and multiple comparisons were also used. Statistical analyses were performed by SPSS® ver 16.0 for Windows®. 

## RESULTS

During a mean period of one-year follow-up, 45 (79%) patients were diagnosed with stable graft function (group A) and 12 (21%) experienced clinical acute rejection episodes (group B). Table 1 summarizes the patients demographics and some clinical characteristics with no statistically significant differences between the stable graft function group (n=45) and the clinical acute rejection group (n=12). Twenty patients received allograft from living-related donors (15 in group A *vs. *five in group B) and 37 patients from living-unrelated donors (30 in group A *vs. *seven in group B). All but four patients received HLA-mismatched renal transplants. The mean serum creatinine levels during the one-year follow-up was slightly lower in patients with stable graft function compared to those with clinical rejection. Moreover, the incidence of post-transplantation CMV infection was not statistically different between groups A and B (Table 1).

**Table 1:** Demographic data and clinical characteristics of the studied patients

Parameters	Stable graft group (n=45)	Acute rejection group (n=12)
Mean±SD age (yrs)	45.2±13.4	41.3±7.5
Female/Male	18/27	6/6
	40%/60%	50%/50%
Number of HLA*-mismatches (HLA-A, B, DR)		
0 mM	3 (7%)	1 (8%)
2 mM	1 (2%)	1 (8%)
3 mM	5 (11%)	0 (0%)
4 mM	9 (20%)	3 (25%)
5 mM	14 (31%)	3 (25%)
6 mM	13 (29%)	4(33%)
CMV† status		
–ve	32 (71%)	9 (75%)
+ve	13 (29%)	3 (25%)
Relation with donors		
No	30 (67%)	7 (58%)
Yes	15 (33%)	5 (42%)
Immunosuppresive regimen		
Cyclosporine A	Adjust dose	Adjust dose
Mycophenolate Mofetil	Adjust dose	Adjust dose
Methyl prednisolone	Adjust dose	Adjust dose
Mean±SD serum Cr‡ levels (mg/dL)		
Day 0	6.52±2.63	7.90 ± 2.26
Day 14	1.57±0.85	1.59 ± 0.76
Day 28	1.59±0.46	1.79 ± 1.20
Week 12	1.59±0.54	1.72 ± 1.01
Week 24	1.43±0.55	1.63 ± 0.82
Week 36	1.60±1.00	1.65 ± 0.94
Week 48	1.86±0.95	1.79 ± 1.01

* HLA: Human leukocyte antigens;

† CMV: Cytomegallovirus;

‡ Cr: Creatinine

Immunological findings 

The frequency of IFN-γ-producing cells and TGF-β contents in supernatants of ELISPOT experiments were measured before transplantation and two weeks, one month and three months following transplantation in patients with stable graft function (n=45) and clinical rejection group (n=12). Similar measurements were made for biopsy- proven rejection group (n=10) only after confirmation of the rejection. 

**Figure 1:**
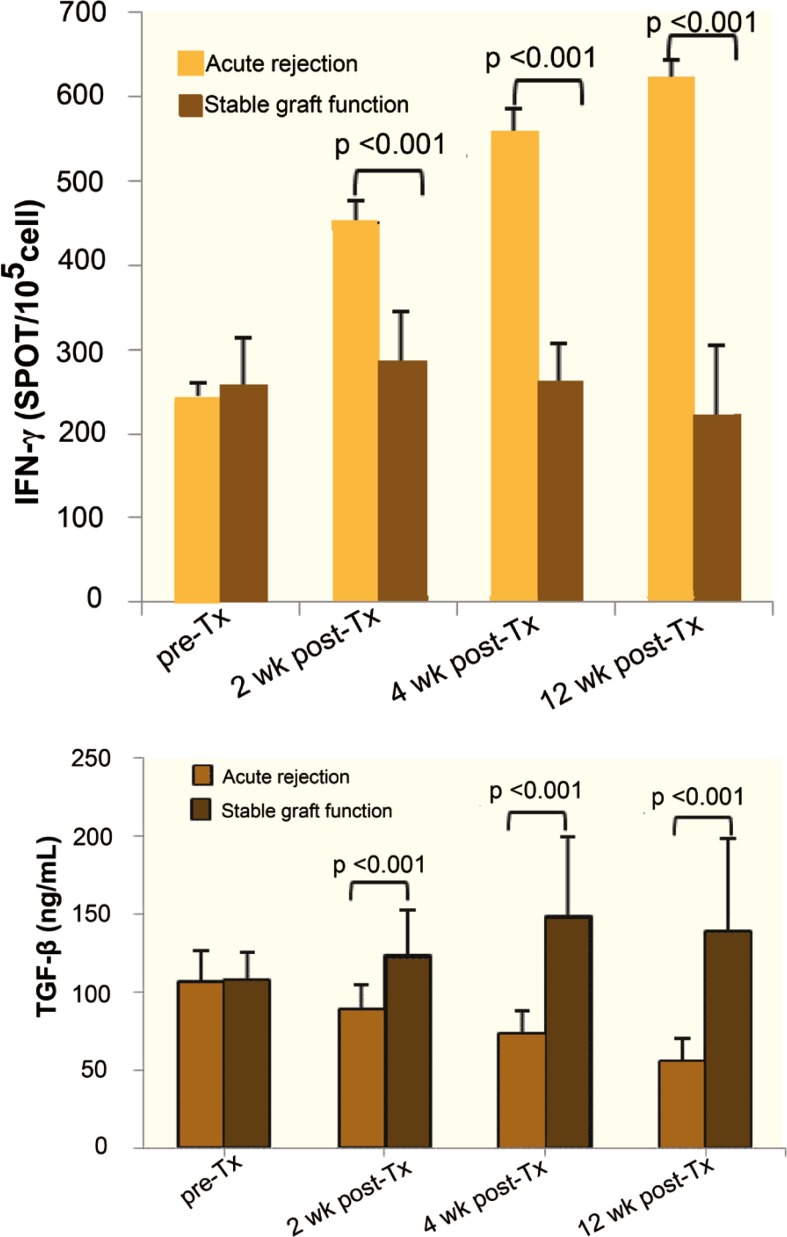
Comparison of the mean frequency of IFN-γ-producing cells and TGF-β contents in supernatants of ELISPOT assay between patients with stable graft function and rejection

The mean frequency of IFN-γ-producing cells in pre-transplantation samples did not show any statistically differences between groups A and B. However, the frequency of these cells was significantly higher in those with rejection at all three times after transplantation (p<0.001, Fig 1). Similarly, the mean levels of TGF-β was not statistically different between groups A and B before transplantation, nonetheless, it was significantly higher in those with stable graft function compared to those who developed rejection post-transplantation (p<0.001, Fig 1).

**Figure 2:**
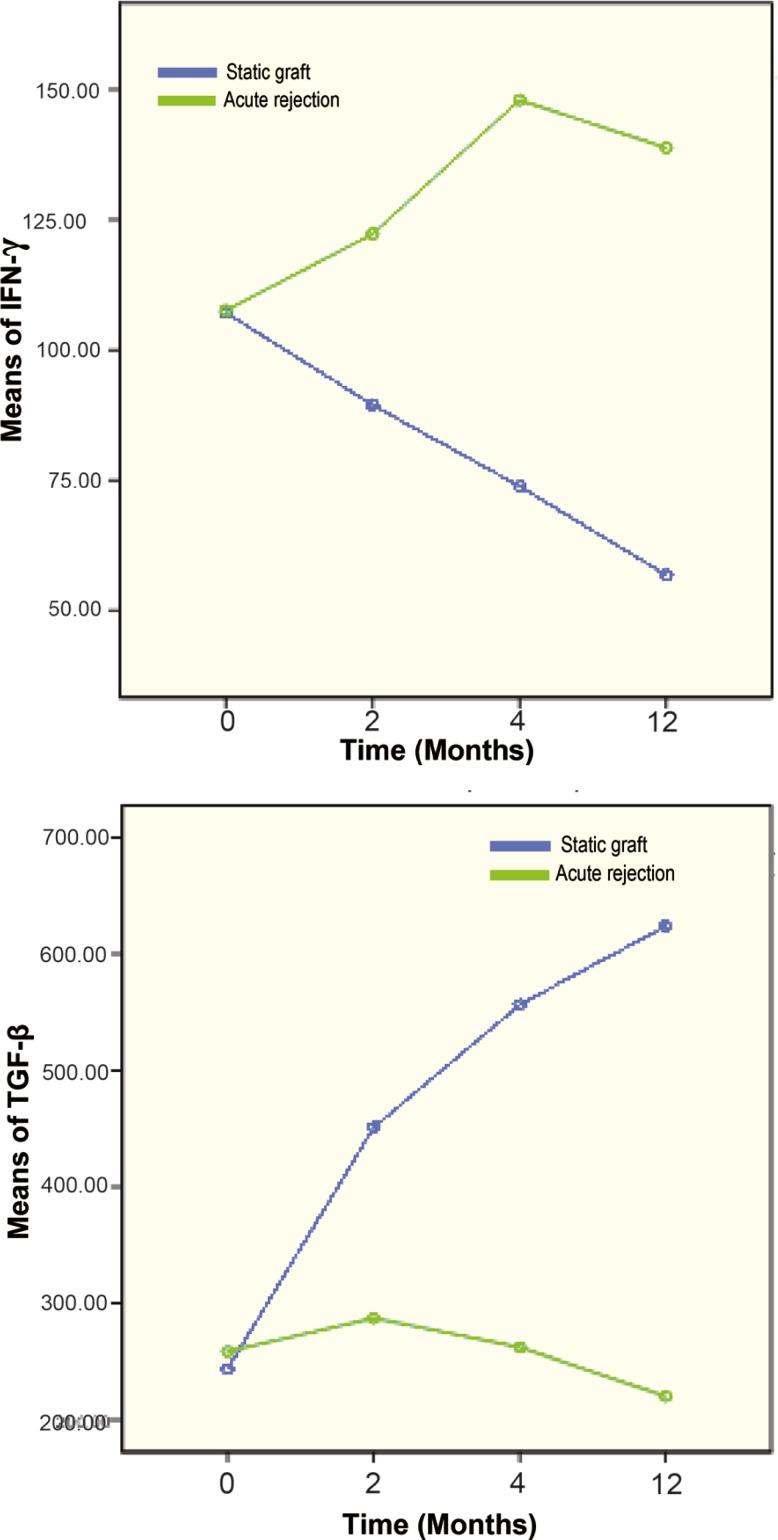
Progressive changes in IFN-γ and TGF-β cytokines in both stable graft and rejection groups

Comparing the post-transplantation levels of TGF-β and the mean numbers of IFN-γ- producing cells between groups A and B demonstrated a continues increase in TGF-β and decrease in the frequency of IFN-γ-producing cells in those with stable graft function compared to patients with clinical rejection (Fig 2). 

**Figure 3:**
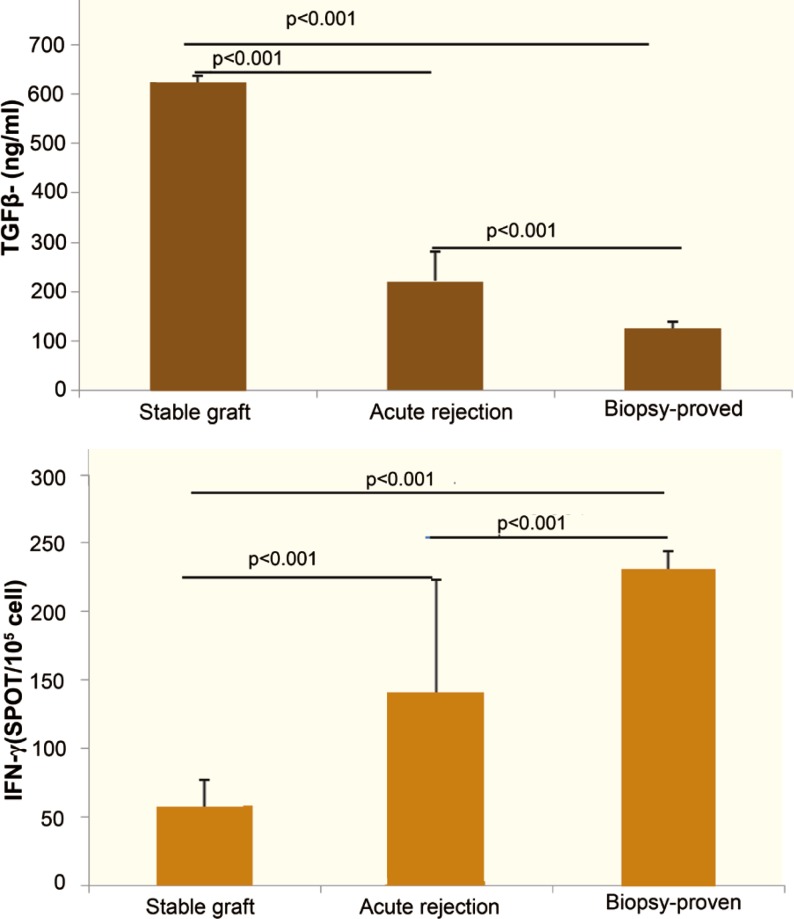
Comparison of the mean frequency of IFN-γ-producing cells and TGF-β contents in supernatants of ELISPOT assay between stable graft function, clinical acute rejection, and biopdy-proven acute rejection groups

The mean frequency of IFN-γ-producing cells in those with biopsy-proven rejection was significantly higher than that in group A (p<0.001) and group B (p<0.001) patients three months post-operatively (Fig 3). In addition, a significant lower concentration of TGF-β was observed in patients with biopsy-proven rejection compared to the other two groups (p<0.001, Fig 3). 

## DISCUSSION

Noninvasive protocols to early diagnosis of patients at risk of graft failure during the post-transplant period are not only important for accurate immune monitoring and timely therapeutic interventions to prevent graft loss, but are also less life-threatening for transplant patients [6, 23]. In spite of general improvements in short-term allograft outcome, acute rejection episodes occur in almost 10% of renal transplant patients within the first post-transplantation year and almost 50% of the patients lose their grafts in ten years after transplantation [4]. The major goal in organ transplantation is to improve long-term graft survival using tolerance induction strategies such as T cells costimulators blockade, mixed chimerism and T cells depletion, which in turn decrease the incidence of rejection episodes and even result in discontinuing of immunosuppressive drugs [4]. 

Our knowledge of the immune mechanisms underlying acute rejection has increased markedly, but the main reason of graft loss attributable to chronic allograft dysfunction still remains to be understood. Various studies have indicated that chronic rejection has a close relationship with acute rejection in early post-transplantation period. Leukocyte infiltration into the graft particularly lymphocytes and macrophages is a well-known characteristic of acute cellular rejection. It has been shown that, T cells, especially helper (CD4+) subsets, are major players in alloimmune responses either through direct or indirect pathways against transplanted organ and subsequently acute rejection. This cell-mediated alloimmune response happens mainly through proinflammatory cytokines and other inflammatory mediators released from activated alloreactive T cells. Therefore, defining the exact role of cytokines in graft rejection could be promising in developing novel strategies to predict and more importantly to prevent graft failure [21, 24]. 

Results of this prospective study suggest that early post-transplantation evaluation of cellular alloimmune response, as assessed by serial ELISPOT measurements of IFN-γ-producing lymphocytes, is significantly predictive for clinical acute rejection in renal transplant patients. We observed that patients with stable graft function had decreased frequencies of IFN-γ-producing cells among stimulated PBMCs with donor cells compared with increased number of these inflammatory cells in patients with clinical and biopsy-proven acute rejection. The stable patients showed a higher concentration of TGF-β, after stimulation of PBMCs with donor cells, compared to those with clinical acute rejection and biopsy-proven rejection. In keeping with our findings, two previous studies on renal transplant patients by Nickel, *et al*, [1] and Hricik, *et al*, [2] showed that patients at risk for immune-mediated graft deterioration or with acute rejection in the first post-transplantation year had higher frequencies of IFN-γ-producing donor-reactive cells in ELISPOT assay compared to non-rejecting patients. However, in contrast with Nickel, *et al*, study we did not observe any significant difference for pre-transplant ELISPOT assay between the stable group and patients with acute rejection episodes. 

Finding the close correlation between immune-mediated graft loss and ELISPOT frequency of IFN-γ-producing donor-reactive cells in biopsy-proven rejection group, as well as clinical rejection group, indicates that cellular alloreactivity assessment by this method could be useful for early categorizing of patients into high and low risk for immune-mediated graft failure. This method would also allow tapering of high dose immunosuppression for low-risk patients and early additional therapeutic interventions for high-risk patients [1, 2]. 

This donor-specific assay of cellular immunity which defined by high production of IFN-γ, a prototype cytokine of Th1 response, in patients at risk of early allograft dysfunction could be used as a simple and reliable early surrogate marker for late transplant outcome [2]. 

Additionally, measuring TGF-β contents in cell culture supernatants of ELISPOT assay confirms the profile of IFN-γ-producing cells with respect to clinical outcome in the three studied groups of patients. Quantification of TGF-β in supernatants of this co-culture (inactivated donor cells *vs*. recipients PBMCs) could be indicative partially for serum levels of this cytokines in relation to allograft function in these patients. We found a higher significant levels of TGF-β in patients with stable graft function compared to other two studied groups of patients with allograft rejection. An almost similar study by Tellioglu, *et al*, [25] on evaluation of serum levels of TGF-β, IL- 10 and IFN-γ in renal transplant patients either with acute rejection or with stable graft function, showed a significant correlation between these cytokines and clinical outcomes of patients in the studied groups. They found that monitoring of cytokine secretion could be used as a promising tool in determining immunological risk in renal transplant patients. However, our data should be interpreted cautiously because higher amount of this pleiotropic cytokine in long-term could be a risk factor for graft vasculopathy and chronic rejection, as it promotes angiogenesis and a profibrotic condition [18-20]. 

In conclusion, serial post-transplantation monitoring of IFN-γ-producing donor reactive cells during the first months is a clinically feasible approach for identification of kidney allogarft recipients at risk of developing immune-mediated graft damage and graft loss. Patients with clinical or biopsy-proved acute rejection had higher frequencies of IFN-γ- producing cells as well as lower contents of TGF-β early after transplantation. This might help to guide the tapering of immunosuppression therapy in patients with lower levels of alloreactivity or increasing immunosupression in those with high levels of allorectivity. Finally, longer follow-up of patients, more immunological tests and further studies with larger sample size are needed to determine the relevance of ELISPOT method with clinical outcomes of renal transplant patients. 
